# Efficacy of single-stage posterior surgery for HIV-positive patients with thoracolumbar tuberculosis

**DOI:** 10.1186/s12981-022-00478-9

**Published:** 2022-11-22

**Authors:** Yao Zhang, Chang-song Zhao, Jia-min Chen, Qiang Zhang

**Affiliations:** 1 Department of Orthopedics, Beijing Ditan Hospital, Capital Medical University, No. 8, Jingshun East Street, Chaoyang District, Beijing, 100015 China; 2 Department of Pathology, Beijing Ditan Hospital, Capital Medical University, No. 8, Jingshun East Street, Chaoyang District, Beijing, 100015 China

**Keywords:** HIV, Thoracolumbar vertebrae, Spinal tuberculosis, Posterior surgery

## Abstract

**Objective:**

We aimed to observe the clinical effect of single-stage posterior surgery on HIV-positive patients with thoracolumbar tuberculosis.

**Methods:**

From October 2015 to October 2019, 13 HIV-positive patients with thoracolumbar tuberculosis who underwent single-stage posterior surgery were retrospectively analyzed (observation group), and 13 HIV-negative patients with thoracolumbar tuberculosis who were matched with the gender, age, operative site, and surgical approach during the same period were selected as the control group. Postoperative complications, hemoglobin, albumin, CD4^+^T lymphocyte count, operative site, operative time, and blood loss were recorded between the two groups. The clinical efficacy was evaluated by the visual analog scale (VAS), American Spinal Injury Association (ASIA) scale, erythrocyte sedimentation rate (ESR), C-reactive protein (CRP), kyphotic angle, correction rate of kyphosis, angle loss, and bone graft fusion time.

**Results:**

In the observation group, 7 patients had postoperative complications, including 1 patient with cerebrospinal fluid leakage, 1 patient with nerve root irritation, 1 patient with an opportunistic infection, and 4 with delayed wound healing. In the control group, 2 patients developed postoperative complications, including 1 with nerve root irritation and 1 with delayed wound healing. There was no statistically significant difference in the incidence of postoperative complications between the two groups (*P* > 0.05). CD4^+^T lymphocyte count, hemoglobin, and albumin in HIV-positive patients with postoperative complications were statistically different from those without postoperative complications (*P* all < 0.05). No tuberculosis recurrence was found at the last follow-up, ESR and CRP returned to normal, and there were no statistically significant differences in bone graft fusion time, VAS score, ASIA scale, correction rate of kyphosis, and angle loss between two groups (*P* all > 0.05).

**Conclusion:**

Single-stage posterior surgery for HIV-positive patients with thoracolumbar tuberculosis could achieve satisfactory clinical efficacy through comprehensive preoperative evaluation, standardized perioperative antiviral and anti-tuberculosis treatments, and prevention of postoperative complications.

## Introduction

Human immunodeficiency virus (HIV) mainly attacks CD4^+^T lymphocytes and causes cellular immune deficiency, and patients with HIV infection may develop acquired immune deficiency syndrome (AIDS). AIDS has become a significant public health problem in China and the world. With the development of antiretroviral therapy (ART), AIDS has changed from a disease with high mortality to a chronic disease. Meanwhile, HIV-infected patients may need surgical or invasive treatment due to some surgical diseases [1].

Studies showed that about 25% (446/1782) of HIV-infected patients would receive surgical treatment in their lifetime, and 7.8% (139/1782) of them would receive grade 3 or above surgery [2]. Another study showed that the proportion of patients undergoing surgery for trauma, infection, tumor, and degenerative dysfunction was 5.9% (29/484), 46.9% (227/484), 23.5% (114/484), and 23.5% (114/484) respectively, among which surgery for infection was the most common [3].

Tuberculosis (TB) infection is one of the most common complications for patients, from early HIV infection to AIDS. Symptoms of TB combined with HIV infection are atypical, especially extrapulmonary tuberculosis. Spinal tuberculosis is the most common and dangerous, and pathological fractures of tuberculosis may cause kyphosis. In recent years, with the increase in HIV infections and drug-resistant strains of TB, spinal tuberculosis has been increasing yearly [4–6]. For surgical methods, there are simple anterior approach, simple posterior approach, and anterior and posterior approach combined with debridement and bone graft fusion. However, the selection of surgical methods is still controversial [7–11]. At the same time, a weakened immune system due to HIV infection could increase the risk of perioperative, including opportunistic infections and various surgical complications such as surgical site infection, sepsis, et al. Hence, risk assessment and the timing of surgical treatment are critical [12–15].

There were few reports on the surgical treatment of HIV-positive spinal tuberculosis patients at home and abroad. In this study, 13 patients with HIV-positive thoracolumbar tuberculosis were treated by single-stage posterior surgery with satisfactory clinical efficacy. The experience of perioperative treatment is summarized as follows.

## Materials and methods

### Demographics, description of the patient population

From October 2015 to October 2019, 13 HIV-positive patients with thoracolumbar tuberculosis were diagnosed and treated in our department (observation group), including 8 males and 5 females. The age ranged from 23 to 66 years, with an average of (44.1 ± 12.9) years. The disease ranged from 4 to 34 months, with an average course of (14.3 ± 8.4) months. For underlying diseases, there were 4 patients with syphilis, 2 patients with hepatitis B, 1 with hepatitis C, 1 with hypertension, and 1 with diabetes. In addition, 6 cases (46.2%) were complicated with neurologic impairment, such as muscle weakness and sensory dysfunction. ASIA scale: 1 case was grade B, 2 cases were grade C, 3 cases were grade D, and 7 cases were grade E. All patients in the observation group had different chest and back pain, fever, emaciation, and fatigue. Preoperative X-ray, Computed tomography (CT), and Magnetic resonance imaging (MRI) were completed, which showed typical vertebral body destruction or collapse in 10 cases (76.9%), sequestration in 11 cases (84.6%), intraspinal epidural or paravertebral abscess in 6 cases (46.2%), and intervertebral space narrowing in 9 cases (69.2%). The kyphotic angle ranged from 13° to 45°, with an average of (24.5 ± 7.9)°. HIV-RNA viral load: 6 patients were target not detected (TND), 3 patients were < 20 copies/ml, and other 3 patients were 207 copies/ml, 94 copies/ml, and 423 copies/ml, respectively.

The control group included 13 HIV-negative patients with thoracolumbar tuberculosis, 9 males and 4 females. The average age was (42.5 ± 12.6) years, from 26 to 64 years. The disease course ranged from 8 to 35 months, and the median was 13.0 (10.5, 19.0) months. Among them were 1 case with syphilis, 1 with hepatitis B, 1 with hypertension, and 1 with type 2 diabetes. Five cases (38.5%) were complicated with neurologic impairment. ASIA scale: 1 case was grade B, 2 cases were grade C, 2 cases were grade D, and 8 cases were grade E. All patients had various degrees of chest and back pain, fever, emaciation, and fatigue. Preoperative imaging findings included typical vertebral body destruction or collapse in 9 cases (69.2%), sequestration in 10 cases (76.9%), intraspinal epidural or paravertebral abscess in 5 cases (38.5%), and intervertebral space narrowing in 9 cases (69.2%). The kyphotic angle ranged from 15° to 46°, with an average of (26.3 ± 8.8)°.

Inclusion criteria were as follows: (1) The diagnosis of HIV/AIDS was according to the diagnostic criteria of the AIDS Diagnosis and Treatment Guide, and CD4^+^T lymphocyte count of patients > 50 cells/ul; (2) Patients were adults with thoracolumbar tuberculosis; (3) Spinal tuberculosis was confirmed by MRI, CT, X-ray, and acid-fast staining or polymerase chain reaction (PCR) in the pathology department during the operation; (4) All patients in this study voluntarily signed an informed consent form to join the scientific research and an informed consent form for surgical treatment. Exclusion conditions: (1) Lesions in patients were more than two segments; (2) Patients had active pulmonary tuberculosis; (3) Patients were over 60 years old with underlying severe diseases; (4) Children and infants were not involved.

### Preoperative preparation

Patients in the observation group selected the highly active antiretroviral therapy (HAART) regimen for HIV infection [16]. Our patients were all treated for tuberculosis in specialized hospitals before coming to our hospital. Furthermore, before surgery, we consulted specialists from our infectious disease department for anti-tuberculosis treatment and followed their suggestions. Currently, the first-line anti-tuberculosis drugs recommended by the International Federation of Antituberculosis and Lung Diseases mainly include isoniazid, rifampicin, pyrazinamide, streptomycin, and ethambutol [17].

Meanwhile, patients' immune and nutritional status was evaluated and improved. For 4 patients with malnutrition in the observation group, the hemoglobin was (90.8 ± 2.8) g/L and (105.0 ± 4.7) g/L before and after nutritional support treatment; albumin was (31.7 ± 2.6) g/L and (37.7 ± 1.7) g/L, respectively. The difference was statistically significant (*P* = 0.012, *P* = 0.032, respectively).

### Surgical techniques

**Fig. 1:**
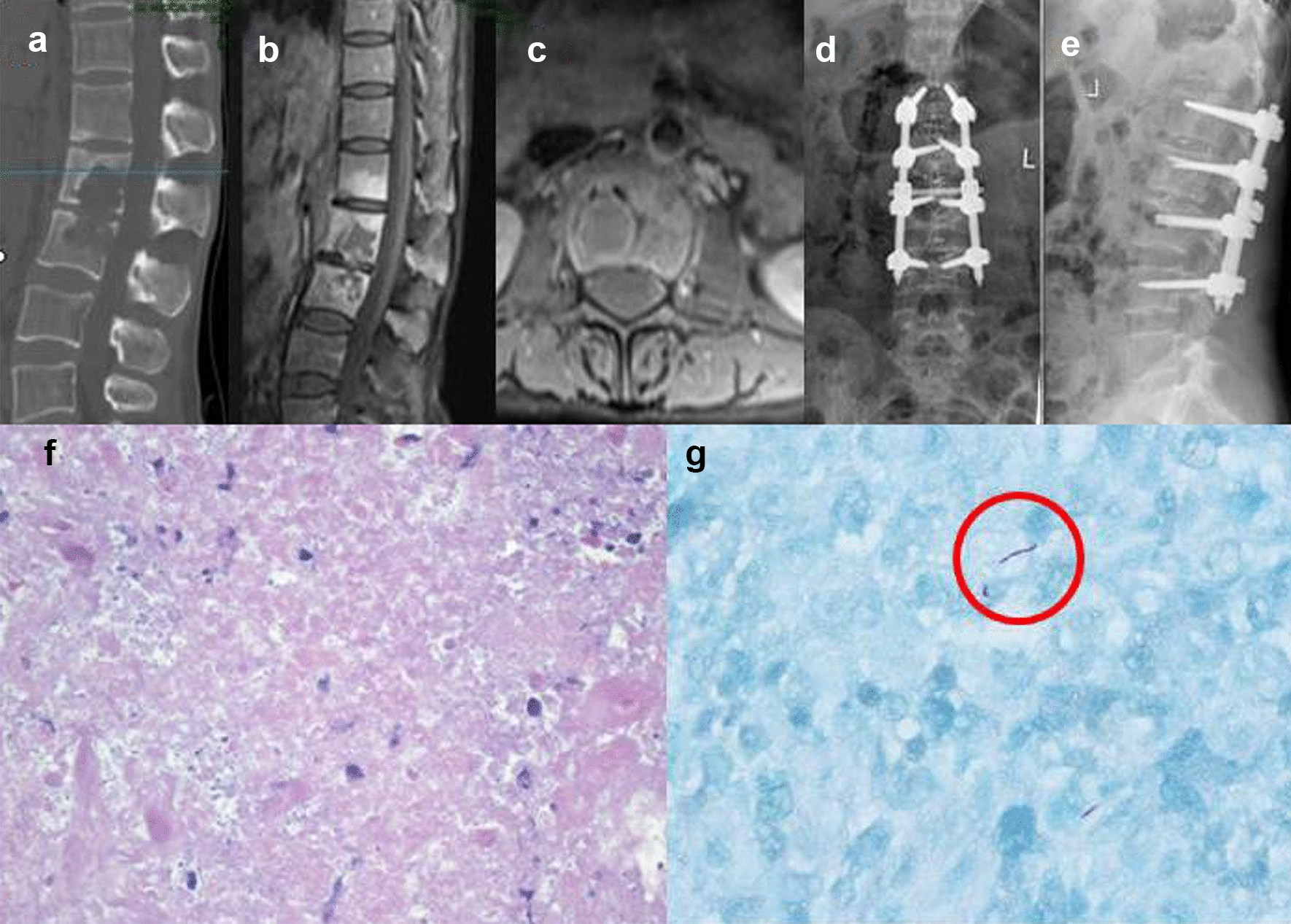
For case No. 3 in the observation group, the patient (female, 45 years old) was admitted to the hospital via the outpatient department with “spinal tuberculosis,” mainly due to “Low back pain with fever and sweating for 2 years, aggravated with walking restriction for 2 months”. The patient was diagnosed with HIV infection 8 years ago and was treated with highly active antiretroviral therapy (HAART). The patient received a diagnosis of tuberculosis 10 years ago and was treated for tuberculosis in specialized hospitals before coming to our hospital. The HIV-RNA viral load of the patient was not detected; CD4^+^T lymphocyte count, 266 cells/ul. Preoperative CT showed L2-3 vertebral destruction with cavity and sequestrum (**a**), preoperative MRIs showed L2-3 vertebral destruction with abscess, hypointense, hyperintense, hyperintense and hyperintense on T1WI, T2WI, STIR and enhanced MRI images, respectively (**b**, **c**), X-ray of postoperative one year showed interbody fusion is in a good position (**d**, **e**), HE staining (×40) showed caseous necrosis, necrotic area structure without cell and nucleus (**f**), Acid-fast staining (×40, as shown in the red circle) showed tuberculosis bacilli (**g**)

**Fig. 2:**
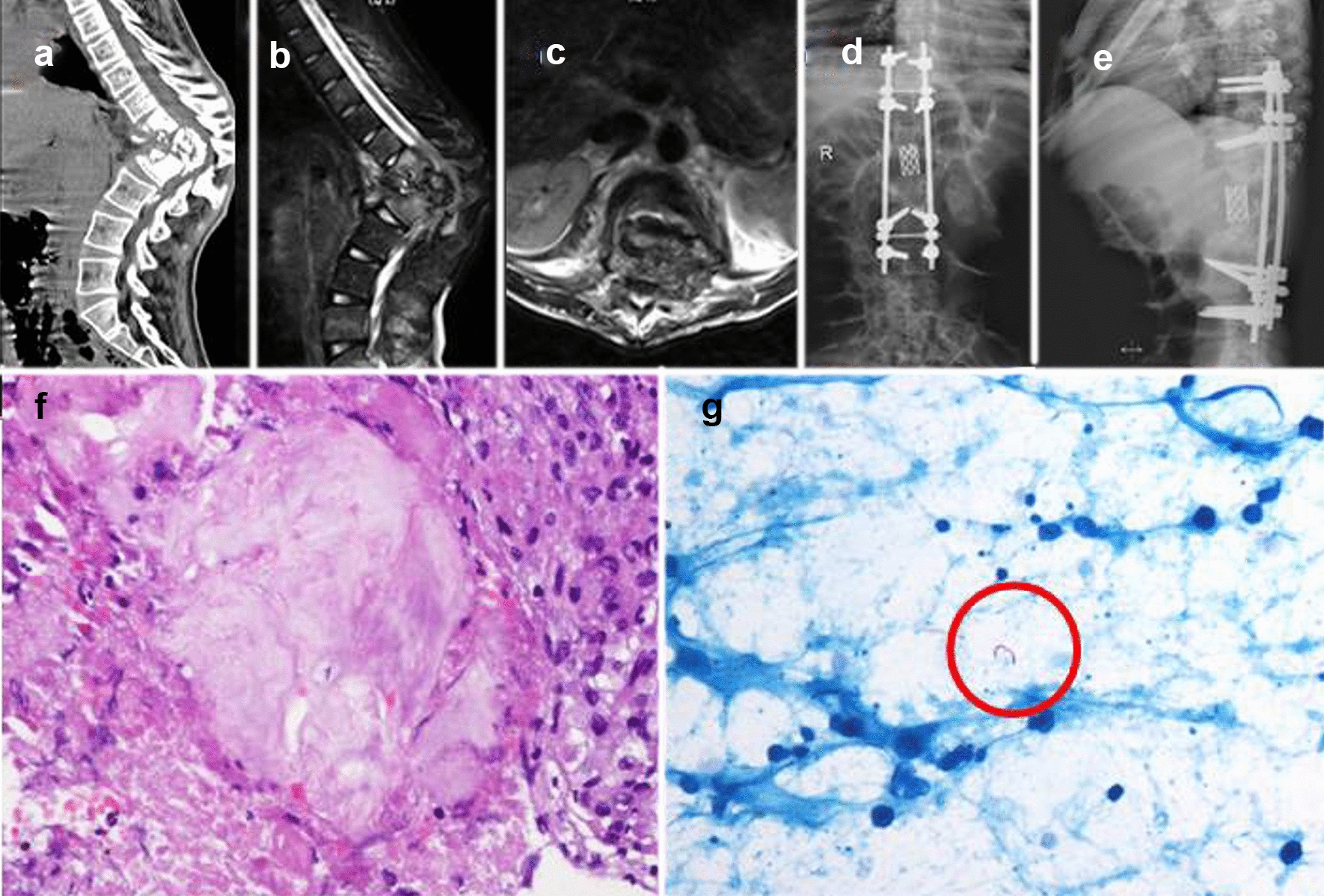
For case No. 2 in the control group, the patient (male, 27 years old) was admitted to the hospital via the outpatient department with “spinal tuberculosis,” mainly due to “Chest and back pain with fever and sweating for 1 year, aggravated with walking restriction for 3 months”. The patient was diagnosed with tuberculosis 5 years ago and was treated for tuberculosis in specialized hospitals before coming to our hospital. Preoperative CT showed T12-L1 vertebral and intervertebral disc destruction with kyphosis and stenosis (**a**), Preoperative MRIs showed abscess break into the spinal canal and dural sac compression seriously (**b**, **c**), X-ray of postoperative two years showed interbody fusion, and correction rate of kyphosis is in a good position with no loosening (**d**, **e**), HE staining (×40) showed caseous necrosis, necrotic area structure without osteogenesis change (**f**), Acid-fast staining (×40, as shown in the red circle) showed tuberculosis bacilli (**g**)

Patients in both groups received single-stage posterior debridement, interbody bone graft fusion, and instrumentation. Under general anesthesia, the patients were placed in a prone position. Extraperiosteal dissection was performed through the posterior median incision to expose the posterior spinal elements, including lamina, facet joint, and transverse process, to 1–2 upper and lower vertebras. Transpedicular screws were placed into the affected vertebrae if the upper and lower portions of the vertebrae had not been destroyed by infection by preoperative symptoms and imaging. The connecting rod was placed and stabilized to avoid spinal cord injury induced by the instability of the spine during decompression and debridement. Following the excision of the necrotic disc, the collapsed vertebrae, and paravertebral abscess, radical debridement was performed until bleeding margins were obtained. After sufficient spinal cord decompression, the kyphosis was corrected by distracting between the adjacent, normal vertebra. We then inserted a suitable autograft or allograft (streptomycin 1.0 g and isoniazid 0.3 g were injected simultaneously) in the prepared bone trough to reconstruct the vertebrae. After no active bleeding was checked, the drainage tube insertion and closing incisions were performed. Finally, the specimens were sent for pathological examination (Figs. 1, 2).

We removed the tube by treatment with antibiotics 3–5 days after surgery, till 24 h drainage < 50 ml. We recommended that patients stay in bed for 1–2 weeks after strengthening nutrition and other supportive therapy.

### Data collection

Hemoglobin, albumin, operative site, operative time, and blood loss were observed and recorded in two groups, and CD4^+^T lymphocyte count was recorded in the observation group. Postoperative complications (orthopedic complications, HIV, and tuberculosis-related complications) were recorded in the two groups. We performed postoperative follow-ups on the 1st, third, sixth, ninth, and 12th months respectively. After 1 year, follow-up was carried out once every 3 or 6 months. Follow-up included blood indicators (such as routine blood tests, liver and kidney function, ESR, CRP, and CD4^+^T lymphocyte count in the observation group). Imaging examination was to observe the bone graft fusion and the outcome of tuberculosis; the VAS score was used to evaluate the relief degree of thoracolumbar and back pain, and the ASIA scale was used for patients with spinal cord nerve function injury (A: Complete: No motor or sensory function is preserved in the sacral segments S4-S5. B: Incomplete: Sensory function is preserved, but no motor function is preserved below the neurological level and includes the sacral segments S4–S5. C: Incomplete: Motor function is preserved below the neurological level, and more than half of key muscles below the neurological level have a muscle grade of less than 3. D: Incomplete: Motor function is preserved below the neurological level, and at least half of key muscles below the neurological level have a muscle grade of 3 or more. E: Normal: Motor and sensory functions are normal). We measured the kyphotic angle for patients with kyphosis. We calculated the correction rate and loss degree of kyphosis [evaluation using correction rate and angle loss: correction rate of kyphosis = (preoperative kyphosis angle − postoperative kyphosis angle)/preoperative kyphosis angle × 100%; angle loss = kyphosis angle at last follow-up − postoperative kyphosis angle].

### Statistical analysis

All statistical analyses were performed using the SPSS software (version 25.0; IBM SPSS, New York, USA). Continuous variables were presented as mean ± SD or medians with interquartile ranges, while categorical variables as the frequencies or percentages of events. Mann–Whitney *U* test was used for nonnormally distributed continuous variables and a *t*-test for normally distributed variables. The *P* value ≤ 0.05 was considered to indicate statistical significance.

## Results

### Outcomes of complications

No significant differences existed between the observation and control groups in hemoglobin, albumin, operative site, operative time, and blood loss (*P* all > 0.05, Table 1).

**Table 1:** Comparison of demographics in patients between HIV-positive patients and HIV-negative patients

	HIV-positive patients (n = 13)	HIV-negative patients (n = 13)	*P *value
Age (years)	44.1 ± 12.9	42.5 ± 12.6	0.761
Sex (male/female)	8/5	9/4	1.000
HB (g/L)	108 ± 8	108 ± 5	0.908
ALB (g/L)	40.1 ± 3.4	38.0 (37.0, 40.5)	0.677
Operative site			
Thoracic vertebrae	2	3	1.000
Thoracolumbar vertebrae	4	4	1.000
Lumbar vertebrae	7	6	1.000
Operative time (min)	212 ± 24	230 (185, 235)	0.938
Blood loss (ml)	540 ± 71	514 ± 73	0.366
Postoperative complications	7	6	0.097
Surgical site infection	0	0	1.000
Delayed healing	4	1	0.332
Opportunistic infections	1	0	1.000
Cerebrospinal fluid leakage	1	0	1.000
Nerve root irritation	1	1	1.000
Dead	0	0	1.000

Continuous variables were presented as mean ± standard deviation (normal distribution) or medians with interquartile ranges (nonnormal distribution). Mann–Whitney *U* test was used for nonnormally distributed continuous variables and a t-test for normally distributed variables

There was no injury to the spinal cord, large vessels, and essential organs, and no death in the two groups. In the observation group, 7 patients developed postoperative complications. One patient suffered postoperative cerebrospinal fluid leakage due to dural tearing while removing lesions. One patient showed symptoms of nerve root stimulation after surgery, which manifested as lower limb pain. In addition, 4 patients had delayed wound healing due to malnutrition, and the other patients had stage I wound healing without chronic sinus formation, cerebrospinal membrane infection, and other complications. One opportunistic infection appeared in a patient with CD4^+^T lymphocyte count < 150 cells/ul. In the control group, 2 patients developed postoperative complications, including 1 with nerve root irritation and 1 with delayed wound healing, without chronic sinus formation, cerebrospinal membrane infection, and other complications.

The incidence of postoperative complications was no statistically different between the observation and control groups (*P* = 0.097, Table 1). Meanwhile, compared with those without complications, there were statistically significant differences in CD4^+^T lymphocyte count, hemoglobin, and albumin in patients with postoperative complications (*P* all < 0.05, Table 2).

**Table 2:** Comparison between HIV-positive patients with or without complications

	with complications (n = 7)	without complications (n = 6)	*P* value
Age (years)	42.4 ± 15.2	46.0 ± 10.8	0.640
Sex (male/female)	4/3	4/2	1.000
CD4^+^ T (cells/ul)	234 ± 53	363 ± 130	0.034
HB (g/L)	102 ± 5	114 ± 5	0.001
ALB (g/L)	37.4 ± 1.4	43.2 ± 2.2	0.000
Operative site			
Thoracic vertebrae	1	1	1.000
Thoracolumbar vertebrae	2	2	1.000
Lumbar vertebrae	4	3	1.000
Operative time (min)	206 ± 24	220 ± 24	0.309
Blood loss (ml)	540 ± 86	540 ± 58	1.000

### Laboratory assessment and function outcomes

In the observation group, the follow-up time was 18–42 months (26.5 ± 6.8) on average, and the time of bone graft fusion was 6–9 months, the median was 8.0 (6.5, 8.5). The follow-up time of the control group ranged from 15 to 39 months (27.9 ± 6.8) months on average, and the time of bone graft fusion ranged from 6 to 8 months, the median was 7.0 (6.0, 8.0). No loosening or fracture of internal fixation was observed in both two groups. There were no significant differences in follow-up time and bone graft fusion time between the two groups (*P* = 0.608, = 0.199, respectively).

In the observation group, the neurological function of all 6 patients recovered after surgery, and the ASIA scale improved by 1–2 grades (Table 3). The symptoms of pain were significantly improved, and the average VAS score was decreased from (7.0 ± 1.1) preoperative to (1.5 ± 0.8) postoperative and 1.0 (0.0, 1.5) at the last follow-up (Table 4). In the control group, the neurological function of all 5 patients recovered after surgery, and the ASIA scale improved by 1–2 grades (Table 3). The symptoms of pain were significantly improved, and the VAS score was decreased from (6.8 ± 0.9) preoperatively to (1.5 ± 1.1) postoperatively and 1.0 (1.0, 2.0) points at the last follow-up (Table 4). There were no significant differences in ASIA scale and VAS score between the two groups (*P* = 1.000, = 0.355, respectively).

**Table 3:** Comparison of ASIA scale between HIV-positive patients and HIV-negative patients

ASIA scale	HIV-positive patients			HIV-negative patients		
	Preoperation	Postoperation	Final follow-up	Preoperation	Postoperation	Final follow-up
A	0	0	0	0	0	0
B	1	1	0	1	1	0
C	2	1	1	2	1	1
D	3	2	1	2	1	1
E	7	9	11	8	10	11

The ASIA scale was used to assess preoperative and postoperative neurologic functions (A: Complete: No motor or sensory function is preserved in the sacral segments S4-S5. B: Incomplete: Sensory function is preserved, but no motor function is preserved below the neurological level and includes the sacral segments S4-S5. C: Incomplete: Motor function is preserved below the neurological level, and more than half of key muscles below the neurological level have a muscle grade of less than 3. D: Incomplete: Motor function is preserved below the neurological level, and at least half of key muscles below the neurological level have a muscle grade of 3 or more. E: Normal: Motor and sensory functions are normal)

**Table 4:** Comparison of laboratory and function parameter between HIV-positive patients and HIV-negative patients

	HIV-positive patients			HIV-negative patients		
	Preoperation	Postoperation	Final follow-up	Preoperation	Postoperation	Final follow-up
ESR (mm/h)	36.0 (22.5, 44.5)	18.5 ± 9.4	8.6 ± 2.8	44.0 (30, 45)	20.4 ± 6.1	8.0 (5.0, 11.0)
CRP (mg/L)	28.9 ± 9.9	9.4 ± 3.9	3.5 ± 1.7	25.6 ± 10.9	11.0 (8.3, 12.6)	3.9 ± 1.8
VAS	7.0 ± 1.1	1.5 ± 0.8	1.0 (0.0, 1.5)	6.8 ± 0.9	1.5 ± 1.1	1.0 (1.0, 2.0)
Kyphotic angle (°)	24.5 ± 7.9	5.2 ± 2.5	7.4 ± 3.3	26.3 ± 8.8	5.6 ± 2.7	7.9 ± 2.9
Correction rate of kyphosis (%)	–	–	71.2 ± 8.9	–	–	68.1 ± 11.1
Angle loss (°)	–	–	2.2 ± 1.4	–	–	2.2 ± 1.3

Continuous variables were presented as mean ± standard deviation (normal distribution) or medians with interquartile ranges (nonnormal distribution). Mann–Whitney *U* test was used for nonnormally distributed continuous variables and a *t*-test for normally distributed variables

In the observation group, there were 8 patients with kyphotic deformity. The kyphotic angle was corrected from an average of (24.5 ± 7.9)° before surgery to (5.2 ± 2.5)° after surgery. The kyphotic angle was (7.4 ± 3.3)° at the last follow-up, the angle loss was (2.2 ± 1.4)°, and the correction rate of kyphosis was (71.2 ± 8.9) % (Table 4). Kyphosis was corrected satisfactorily. In the control group, 7 patients with kyphosis, the kyphotic angle was corrected from an average of (26.3 ± 8.8)° before surgery to (5.6 ± 2.7)° after surgery. The kyphotic angle at the last follow-up was (7.9 ± 2.9)°, and the angle loss was (2.2 ± 1.3)°. The correction rate of kyphosis was (68.1 ± 11.1) % (Table 4). There were no significant differences in correction rate of kyphosis and angle loss between the two groups (*P* = 0.445, = 1.000, respectively).

At the last follow-up, there was no recurrence of tuberculosis in both groups, and ESR and CRP returned to normal. In the observation group, the ESR decreased from 36.0 (22.5, 44.5) mm/h preoperatively to (18.5 ± 9.4) mm/h postoperatively and was (8.6 ± 2.8) mm/h at the last follow-up. CRP was decreased from (28.9 ± 9.9) mg/L preoperatively to (9.4 ± 3.9) mg/L postoperatively and from (3.5 ± 1.7) mg/L at the last follow-up (Table 4). In the control group, the ESR decreased from 44.0 (30, 45) mm/h preoperatively to (20.4 ± 6.1) mm/h postoperatively and was 8.0 (5.0, 11.0) mm/h at the last follow-up. CRP was decreased from (25.6 ± 10.9) mg/L preoperatively to 11.0 (8.3, 12.6) mg/L postoperatively and from (3.9 ± 1.8) mg/L at the last follow-up (Table 4). There were no significant differences in ESR and CRP between the two groups (*P* = 0.600, = 0.575, respectively).

## Discussion

According to the WHO Global Tuberculosis Report 2020 [18], an estimated 10 million people (8.9–11 million) were infected with tuberculosis in 2019. Tuberculosis is one of the most critical complications of HIV/AIDS. Previous studies have reported that the infection rate of HIV with tuberculosis was 25.20% [19], and some studies have reported up to 50% of HIV patients with tuberculosis [20].

Currently, there are no global or national epidemiological studies on spinal tuberculosis. However, it was found that spinal tuberculosis accounted for 69.11% of osteoarticular tuberculosis [6, 21], and elderly patients over 60 years accounted for 44.08%. 24.30% of the patients were 20–39 years old. 2.34% of patients were younger than 20 years old. Shi et al. [22] suggested that the most common site of spinal tuberculosis was the thoracic vertebra and lumbar vertebra, accounting for 47.47% and 59.57 respectively.

Bakhsh et al. [23] reported that the cure rate of spinal tuberculosis, especially in the early stage, could reach 85% with drug therapy alone, but the indications are relatively narrow, and kyphosis and other conditions may occur in the late stage, requiring surgical treatment. At present, it has been widely accepted that surgery should be performed timely in the treatment of spinal tuberculosis. Researchers reported that surgical treatment at the right time relieves pain quickly and can clear lesions and correct deformities. Unfortunately, there were few reports on the surgical treatment of HIV-positive spinal tuberculosis patients [24]. Risk assessment of surgical treatment and the choice of surgical timing is critical [12–15].

In this study, the nutritional status of patients in the two groups was evaluated preoperatively according to the clinical malnutrition assessment criteria. After nutritional support treatment, hemoglobin and albumin in 4 patients in the observation group were significantly increased. No statistical differences existed between the observation and control groups in hemoglobin, albumin, operative site, operative time, and blood loss. In the observation group, 7 patients had postoperative complications, including 4 with delayed wound healing due to malnutrition. In the control group, only 2 patients developed complications. However, there were no statistically significant differences in the incidence of postoperative complications between the two groups. At the same time, there were statistically significant differences in the count of CD4^+^T lymphocytes, hemoglobin, and albumin in the observation group with postoperative complications compared with those without complications. Nutritional support is vital in the perioperative management of HIV patients. Therefore, proper nutritional support, such as supplementing amino acids, albumin, and blood transfusion, is necessary.

In this study, one opportunistic infection, candida Albicans occurred in a patient with CD4^+^T lymphocyte count < 150 cells/ul. Due to surgical stress, the cellular immune function of HIV-positive patients may be impaired. When CD4^+^T lymphocyte count < 200 cells/ul, the probability of fatal opportunistic infections increases significantly [25]. We should evaluate the risk of surgery by combining the level of CD4^+^T lymphocytes before surgery. Non-healing wounds and infection may occur when CD4^+^T lymphocytes are lower than 200 cells/ul, leading to severe consequences [12, 26–28]. Therefore, patients undergoing surgery should receive antiviral therapy before surgery.

So far, the commonly used surgical treatment methods include the anterior approach, posterior approach, anterior and posterior approach combined with debridement, and bone graft fusion. However, there were still controversies about surgical methods [7–11].

Since spinal tuberculosis mainly involves the anterior and medial columns of the vertebral body, the anterior approach has apparent advantages in exposing the surgical field and directly entering the lesion site. Therefore, the anterior approach for debridement, bone graft, and instrumentation is primarily used in treating thoracolumbar tuberculosis and has achieved sound therapeutic effects. However, the anterior approach also has certain disadvantages. In addition to extensive exposure to lesions, cutting off a large number of initially healthy tissues is necessary, resulting in more incredible trauma than the posterior approach, which may result in relatively more complications [29]. At the same time, correcting severe kyphosis by anterior approach is often challenging to achieve the same satisfying effect as the posterior approach and may even lead to the decline of spinal stability after decompression [30, 31].

With the recent improvement of MRI, CT, and ultrasound techniques, posterior surgery has gradually achieved satisfactory results in debridement. At the same time, because of the excellent effect of posterior instrumentation in the correction of kyphosis, the choice of posterior surgery for spinal tuberculosis is more common. In contrast, the posterior approach has a simple anatomical structure, short operative time, less intraoperative blood loss, and a significantly reduced possibility of anterior organ injury. As the most common technique in spinal surgery, it is much easier to master this technique with the assistance of an X-ray or surgical navigation system than to be familiar with the complex anatomical relationship between the anterior approach and the risk of injury to vital organs, blood vessels, and nerves in the thoracic and abdominal cavity. Lee et al. [32] proposed that posterior debridement, bone graft fusion, and instrumentation are effective methods for treating thoracolumbar tuberculosis. Zhang et al. [33–36] reported the clinical efficacy of single-stage posterior debridement and interbody fusion and instrumentation in treating thoracolumbar spinal tuberculosis in several studies, believing that this operation has the advantages of minor trauma, good orthopedic effect, and good efficacy. Zhao et al. [37] believed that compared with the anterior approach, the posterior approach could better correct kyphosis. The most significant disadvantage of this method is that the lesion is not cleared, and the firmness of the bone graft is not as good as that of the anterior approach. Zeng et al. [38] reviewed 177 cases of thoracolumbar tuberculosis. They pointed out that the correction rate of kyphosis and spinal stability of the posterior approach was significantly better than that of the anterior approach, comparable to that of the combined anterior and posterior approach. In addition, the amount of intraoperative blood loss and operative time were significantly less/shorter than that of the anterior and combined anterior and posterior approaches, suggesting that the posterior approach is superior to the other two methods and plays an essential role in thoracolumbar tuberculosis.

In this study, 26 patients with thoracolumbar tuberculosis underwent single-stage posterior debridement, interbody bone graft fusion, and instrumentation. Symptoms in patients had significantly improved, and recovery of neurological function and correction rate of kyphosis was satisfactory. Long-term follow-up showed good bone graft fusion, no significant angle loss, and no tuberculosis recurrence. In addition, there were no significant differences in the time of bone graft fusion, VAS score, ASIA scale, correction rate of kyphosis, and angle loss between the two groups. Therefore, we believe that the posterior approach is safe, effective, and feasible in treating HIV-positive thoracolumbar tuberculosis. However, the sample size of this study is small, which requires further observation and clinical evaluation of long-term efficacy with large sample size.

## Conclusions

We believe that single-stage posterior surgery for HIV-positive patients with thoracolumbar tuberculosis could achieve satisfactory clinical efficacy through comprehensive preoperative evaluation, standardized perioperative antiviral and anti-tuberculosis treatment, and postoperative prevention of complications.

## Data Availability

All data generated or analyzed during this study are included in this published article.

## Abbreviations

AIDS: Acquired immune deficiency syndrome
HIV: Human immunodeficiency virus
VAS: Visual analog scale
ASIA: American Spinal Injury Association scale
ESR: Erythrocyte sedimentation rate
CRP: C-reactive protein
CT: Computed tomography
MRI: Magnetic resonance imaging
HAART: Highly active antiretroviral therapy
ART: Antiretroviral therapy
TB: Tuberculosis

## References

[1] Ryom L, Cotter A, De Miguel R, Béguelin C, Podlekareva D, Arribas JR (2020). 2019 Update of the European AIDS Clinical Society Guidelines for treatment of people living with HIV version 10.0. HIV Med.

[2] Wang L, Chen Y, Wang Y, Liu J, Wen Z, Chen H (2020). Lung cancer surgery in HIV-infected patients: an analysis of postoperative complications and long-term survival. Thorac Cancer.

[3] Shmakova A, Germini D, Vassetzky Y (2020). HIV-1, HAART and cancer: a complex relationship. Int J Cancer.

[4] García-Rodríguez JF, Álvarez-Díaz H, Lorenzo-García MV, Mariño-Callejo A, Fernández-Rial Á, Sesma-Sánchez P (2011). Extrapulmonary tuberculosis: epidemiology and risk factors. Enferm Infecc Microbiol Clin.

[5] O'Son L, Hulland E, Cookson ST, Castro KG, Yaacoub H (2020). Epidemiology and risk factors for extrapulmonary tuberculosis in Lebanon. Int J Tuberc Lung Dis.

[6] Dahlan RH, Ompusunggu SE, Gondowardojo YRB, Priambodo R, Anugerah SW (2022). Spinal tuberculosis: a case series and a literature review. Surg Neurol Int.

[7] Rajasekaran S (2012). Kyphotic deformity in spinal tuberculosis and its management. Int Orthop.

[8] Rajasekaran S, Kanna RM, Shetty AP (2015). History of spine surgery for tuberculous spondylodiscitis. Unfallchirurg.

[9] Shi JD, Wang Q, Wang ZL (2014). Primary issues in the selection of surgical procedures for thoracic and lumbar spinal tuberculosis. Orthop Surg.

[10] Yang P, Zang Q, Kang J, Li H, He X (2016). Comparison of clinical efficacy and safety among three surgical approaches for the treatment of spinal tuberculosis: a meta-analysis. Eur Spine J.

[11] Ruparel S, Tanaka M, Mehta R, Yamauchi T, Oda Y, Sonawane S (2022). Surgical management of spinal tuberculosis-the past, present, and future. Diagnostics (Basel).

[12] Zhao R, Ding R, Zhang Q (2021). What are the risk factors for surgical site infection in HIV-positive patients receiving open reduction and internal fixation of traumatic limb fractures? A retrospective cohort study. AIDS Res Hum Retroviruses.

[13] Aird J, Noor S, Lavy C, Rollinson P (2011). The effect of HIV on early wound healing in open fractures treated with internal and external fixation. J Bone Joint Surg Br.

[14] Howard NE, Phaff M, Aird J, Wicks L, Rollinson P (2013). Does human immunodeficiency virus status affect early wound healing in open surgically stabilised tibial fractures? A prospective study. Bone Joint J.

[15] Wijesekera MP, Graham SM, Lalloo DG, Simpson H, Harrison WJ (2016). Fracture management in HIV positive individuals: a systematic review. Int Orthop.

[16] Brojan LEF, Marca LM, Dias FA, Rattmann YD (2020). Antiretroviral drug use by individuals living with HIV/AIDS and compliance with the Clinical Protocol and Therapy Guidelines. Einstein (Sao Paulo).

[17] Fernandes GFS, Thompson AM, Castagnolo D, Denny WA, Dos Santos JL (2022). Tuberculosis drug discovery: challenges and new horizons. J Med Chem.

[18] World Health Organization (2020). Global tuberculosis report.

[19] da Silva Escada RO, Velasque L, Ribeiro SR, Cardoso SW, Marins LMS, Grinsztejn E (2017). Mortality in patients with HIV-1 and tuberculosis co-infection in Rio de Janeiro, Brazil—associated factors and causes of death. BMC Infect Dis.

[20] McLachlan AJ, Arulventhan R (2013). Extrapulmonary tuberculosis–three cases in the spine. Aust Fam Physician.

[21] Ellis H (2012). Percival Pott; Pott's fracture, Pott's disease of the spine, Pott's paraplegia. J Perioper Pract.

[22] Shi T, Zhang Z, Dai F, Zhou Q, He Q, Luo F (2016). Retrospective study of 967 patients with spinal tuberculosis. Orthopedics.

[23] Bakhsh A (2010). Medical management of spinal tuberculosis: an experience from Pakistan. Spine (Phila Pa 1976).

[24] Magis-Escurra C, Günther G, Lange C, Alexandru S, Altet N, Avsar K (2017). Treatment outcomes of MDR-TB and HIV co-infection in Europe. Eur Respir J.

[25] Visseaux B, Charpentier C, Rouard C, Fagard C, Glohi D, Tubiana R (2014). HIV-2 X4 tropism is associated with lower CD4+ cell count in treatment-experienced patients. AIDS.

[26] Deneve JL, Shantha JG, Page AJ, Wyrzykowski AD, Rozycki GS, Feliciano DV. CD4 count is predictive of outcome in HIV-positive patients undergoing abdominal operations. Am J Surg. 2010;200:694–9 10.1016/j.amjsurg.2010.07.030 21146004

[27] Guild GN, Moore TJ, Barnes W, Hermann C (2012). CD4 count is associated with postoperative infection in patients with orthopaedic trauma who are HIV positive. Clin Orthop Relat Res.

[28] Su J, Tsun A, Zhang L, Xia X, Li B, Guo R (2013). Preoperative risk factors influencing the incidence of postoperative sepsis in human immunodeficiency virus-infected patients: a retrospective cohort study. World J Surg.

[29] He Q, Xu J (2013). Transpedicular closing wedge osteotomy in the treatment of thoracic and lumbar kyphotic deformity with different etiologies. Eur J Orthop Surg Traumatol.

[30] Yao R, McLachlin SD, Rasoulinejad P, Gurr KR, Siddiqi F, Dunning CE (2016). Influence of graft size on spinal instability with anterior cervical plate fixation following in vitro flexion-distraction injuries. Spine J.

[31] Jain AK, Dhammi IK, Jain S, Kumar J (2010). Simultaneously anterior decompression and posterior instrumentation by extrapleural retroperitoneal approach in thoracolumbar lesions. Indian J Orthop.

[32] Lee SH, Sung JK, Park YM (2006). Single-stage transpedicular decompression and posterior instrumentation in treatment of thoracic and thoracolumbar spinal tuberculosis: a retrospective case series. J Spinal Disord Tech.

[33] Zhang H, Huang S, Guo H, Ge L, Sheng B, Wang Y (2012). A clinical study of internal fixation, debridement and interbody thoracic fusion to treat thoracic tuberculosis via posterior approach only. Int Orthop.

[34] Zhang H, Sheng B, Tang M, Guo C, Liu S, Huang S (2013). One-stage surgical treatment for upper thoracic spinal tuberculosis by internal fixation, debridement, and combined interbody and posterior fusion via posterior-only approach. Eur Spine J.

[35] Tang MX, Zhang HQ, Wang YX, Guo CF, Liu JY (2016). Treatment of spinal tuberculosis by debridement, interbody fusion and internal fixation via posterior approach only. Orthop Surg.

[36] Zhang H, Guo Q, Wang Y, Guo C, Tang M (2018). The efficiency of the posterior-only approach using shaped titanium mesh cage for the surgical treatment of spine tuberculosis in children: a preliminary study. J Orthop Surg (Hong Kong).

[37] Zhao C, Luo L, Liu L, Li P, Liang L, Gao Y, et al. Surgical management of consecutive multisegment thoracic and lumbar tuberculosis: anterior-only approach vs posterior-only approach. J Orthop Surg Res. 2020;15:343. 10.1186/s13018-020-01876-3 PMC7441607 32819392

[38] Zeng Y, Cheng P, Tan J, Li Z, Chen Y, Li LT (2019). Comparison of three surgical approaches for thoracolumbar junction (T12–L1) tuberculosis: a multicentre, retrospective study. BMC Musculoskelet Disord.

